# Supplementary material for: The Procaine-Based ProcCluster® Impedes the Second Envelopment Process of Herpes Simplex Virus Type 1
Source: Int J Mol Sci. 2025 Jul 25;26(15):7185. doi: 10.3390/ijms26157185 (PMC12346171; doi:10.3390/ijms26157185)

Figure S1: (a) PC treatment shows no effect on the expression of HSV-1 proteins. RPE-1 cells were infected with HSV-1 at an MOI of 1.0 for 1 h and were subsequently incubated in the presence or absence of 2.5 mM PC. At 24 h p.i., cells were lysed and immunoblots were used to analyze HSV-1 protein expression of icp0, icp8, and gD together with the loading controls α-tubulin or HSP90. All immunoblots are representative of at least three independent experiments. (b) RPE-1 cells were infected with HSV-1 at an MOI of 1.0 for 1 h and were further incubated in the presence and absence of PC. At 24 h p.i., supernatants were collected and progeny virus titers were determined by $TCID_{50}$ titrations. To determine cell-associated virus concentrations, the infectious supernatant was replaced with fresh DMEM with 5% FBS. Subsequently, cells were subjected to three freeze-thaw cycles at -80°C in cell culture plates and the resulting lysates were used for titration after centrifugation for 5 min at 3,000 rpm. The mean + SD of three independent experiments including two biological replicates is depicted. Statistical significance compared to the solvent control was analyzed by unpaired two-tailed t-test (* $p=0.0464$; ns not significant). (c) The gD fluorescence signals were quantified based on MIPs. Z-stacks were maximum intensity projected and mean fluorescence intensities per image were determined. Three experiments with four technical replicates and three images per replicate were analyzed using FIJI's inbuilt measure function. The median mean fluorescence intensity per image was determined for each technical replicate, combined to one mean value for each experiment and depicted as means (± SD). Statistical significance was analyzed by unpaired two-tailed t-test (** $p<0.001$; ns, not significant).

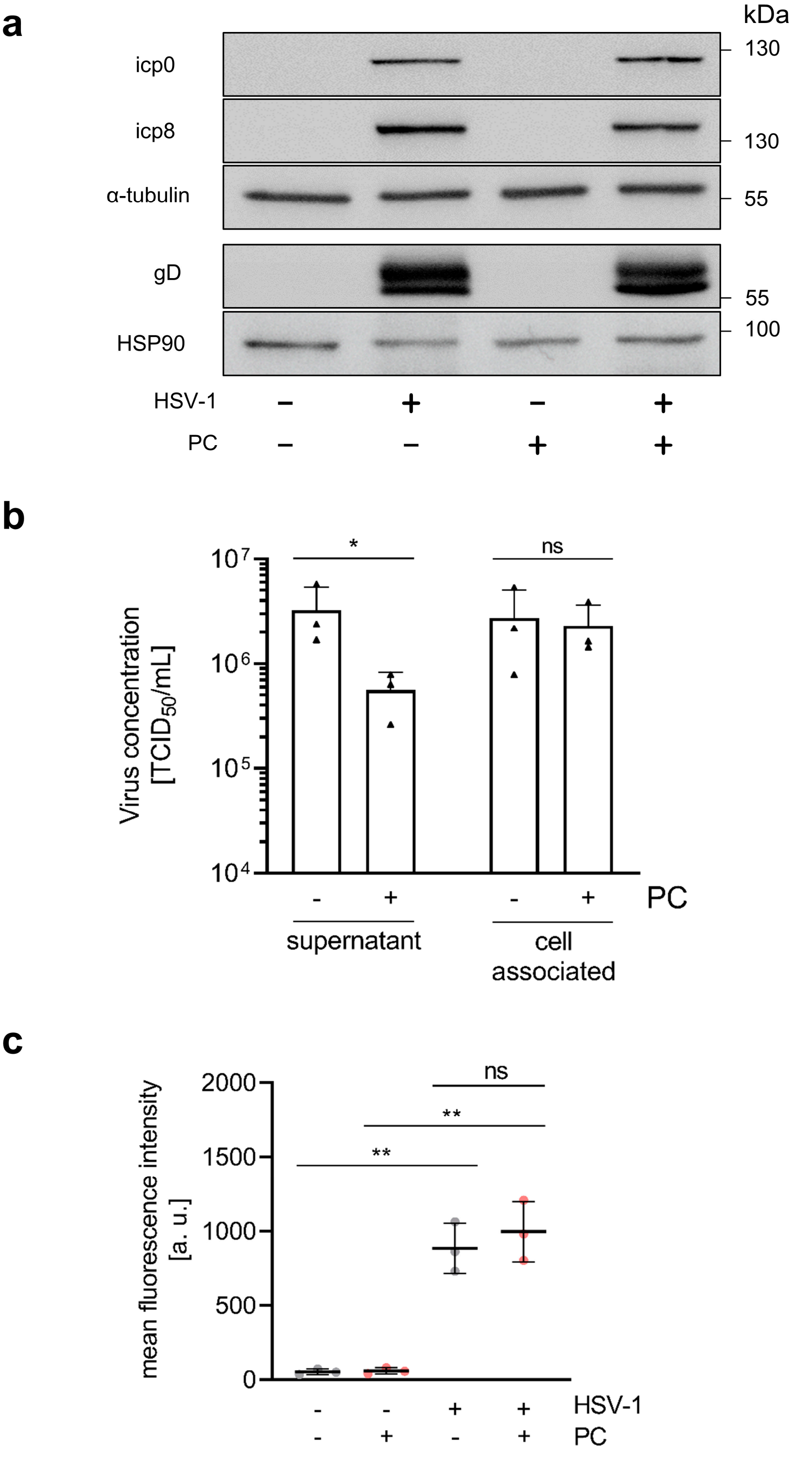
a
kDa
icp0
icp8
α-tubulin
gD
HSP90
130
130
55
55
100
HSV-1
PC
b
Virus concentration [TCID50/mL]
*
ns
supernatant
cell associated
PC
c
mean fluorescence intensity [a. u.]
ns
**
**
HSV-1
PC

Figure S2: PC treatment increases correlation and co-occurrence coefficients of Rab5 as well as Rab11, but not those of Rab7 or LAMP1, with HSV-1 glycoprotein gD. The blue channel (nuclei) was processed to receive ROIs based on Voronoi diagrams to approximate cell shapes. Then, Pearson's and Manders' thresholded coefficients were determined per ROI. For Manders´, both thresholded coefficients, M1, the fraction of gD signal that co-occurs with POI signal, and M2, the fraction of POI signal that co-occurs with gD signal, are shown. In total, three images per condition and experiment (exp) were analyzed and depicted as the median with interquartile range.

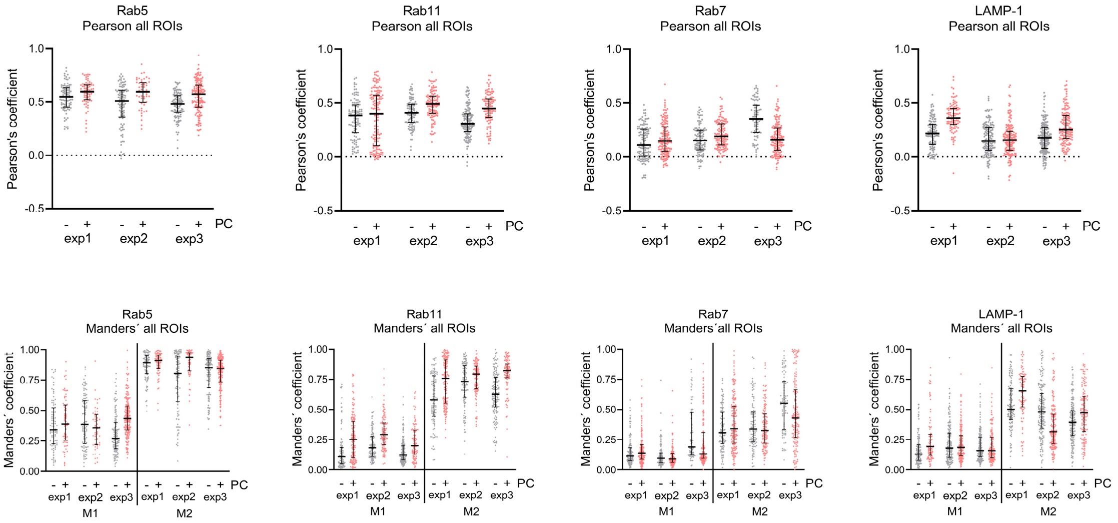

Supplement: Supplementary file 1 [file ijms-26-07185-s001.zip › ijms-3732503-supplementary.pdf]
